# Clinically suspected acute myopericarditis with cardiac tamponade associated with peripheral blood eosinophilia presenting in early pregnancy: a case report

**DOI:** 10.1186/1752-1947-7-129

**Published:** 2013-05-13

**Authors:** Yu Kasamatsu, Takashi Kida, Mayumi Shigeru, Toru Tagashira, Naoki Murai, Eiji Takai, Hideyuki Takaoka

**Affiliations:** 1 Department of Internal Medicine, Matsushita Memorial Hospital, 5-55, Sotojimacho, Moriguchi-shi, Osaka 570-8540, Japan; 2 Department of Cardiovascular Medicine, Takatsuki General Hospital, 1-3-13, Kosobecho, Takatsuki-shi, Osaka 569-1192, Japan

**Keywords:** Cardiac tamponade, Eosinophil, Myopericarditis, Prednisolone, Pregnancy

## Abstract

**Introduction:**

The clinical presentation of eosinophilic myocarditis may vary from asymptomatic to the manifestation of severe symptoms, including cardiac tamponade and arrhythmias. In pregnant patients with this condition, drugs must be used cautiously up to approximately the 4th month of pregnancy because drug use should be limited during the period of fetal organogenesis.

**Case presentation:**

A 30-year-old Asian woman at 14 weeks of pregnancy with progressive malaise was hospitalized. The electrocardiogram revealed ST elevation and low QRS voltage. Echocardiography revealed massive pericardial effusion and myocardial swelling. A laboratory examination revealed an increase in her white blood cell count, with a predominance of neutrophils. Pericardial drainage was performed for relief of the cardiac tamponade. The pericardial effusion revealed an abundance of eosinophils. Subsequently, the peripheral blood eosinophil count began to rise, and the patient was clinically diagnosed with eosinophilic myopericarditis. The patient’s condition improved rapidly following the initiation of prednisolone treatment, and she finally delivered a full-term normal infant.

**Conclusions:**

A patient with clinically suspected myopericarditis in the early stage of pregnancy who improved rapidly with pericardial drainage and prednisolone therapy, and successfully delivered a normal full-term infant; the diagnosis was made in the early stage of the disease, based on the detection of an abundance of eosinophils in the pericardial effusion preceding the subsequent development of peripheral blood eosinophilia.

## Introduction

Eosinophilic myocarditis is characterized by myocarditis associated with marked eosinophilic infiltration of the myocardium and peripheral blood eosinophilia. In addition, the clinical presentation of myocarditis may vary from asymptomatic to the manifestation of severe symptoms [1]. Some patients develop cardiac tamponade due to the associated pericarditis, whereas others may show rapid progression of myocardial necrosis, manifesting as fatal arrhythmias, cardiac arrest, or a clinical state necessitating mechanical assistance for the maintenance of circulation. The mortality associated with the acute stage of this disease is reported to be 7% [2].

In pregnant patients with this condition, drugs must be used cautiously up to approximately the 4th month of pregnancy (the period of fetal organogenesis) because drug use should be limited during this period [3]. We encountered a patient with acute clinically suspected myopericarditis who presented, at 14 weeks of pregnancy, with cardiac tamponade and marked peripheral blood eosinophilia. Here we present this case.

## Case presentation

A 30-year-old Asian woman who was 14 weeks and 5 days pregnant (gravida, 1; para, 0) presented to us with generalized malaise and facial edema in June 2007. She had no significant past medical history or history of allergy. She was a non-smoker and gave no history of drug abuse or eating raw food. She visited a local medical clinic, where a routine blood examination revealed leukocytosis. As the malaise worsened, the patient was brought by ambulance to our hospital for further management. Analysis of her vaginal secretions and ultrasonography of the fetus conducted by an obstetrician/gynecologist revealed no major abnormalities. Pregnancy-induced hypertension was ruled out at that time. Therefore, the patient was referred to the Department of Cardiovascular Medicine of our hospital.

The findings on physical examination at admission were: height, 155cm; weight, 68.2kg; blood pressure, 90/70mmHg. Sinus tachycardia (heart rate, 140/minute; regular beats) was noted, with a body temperature of 36.7°C. The peripheral arterial oxygen saturation was 96% on room air. There were no evident cutaneous eruptions. Jugular venous distention, puffiness of the eyelids and pitting edema of the extremities were noted. Although no significant murmur and rale could be appreciated on chest examination, a pericardial rub was heard.

**Figure 1:**
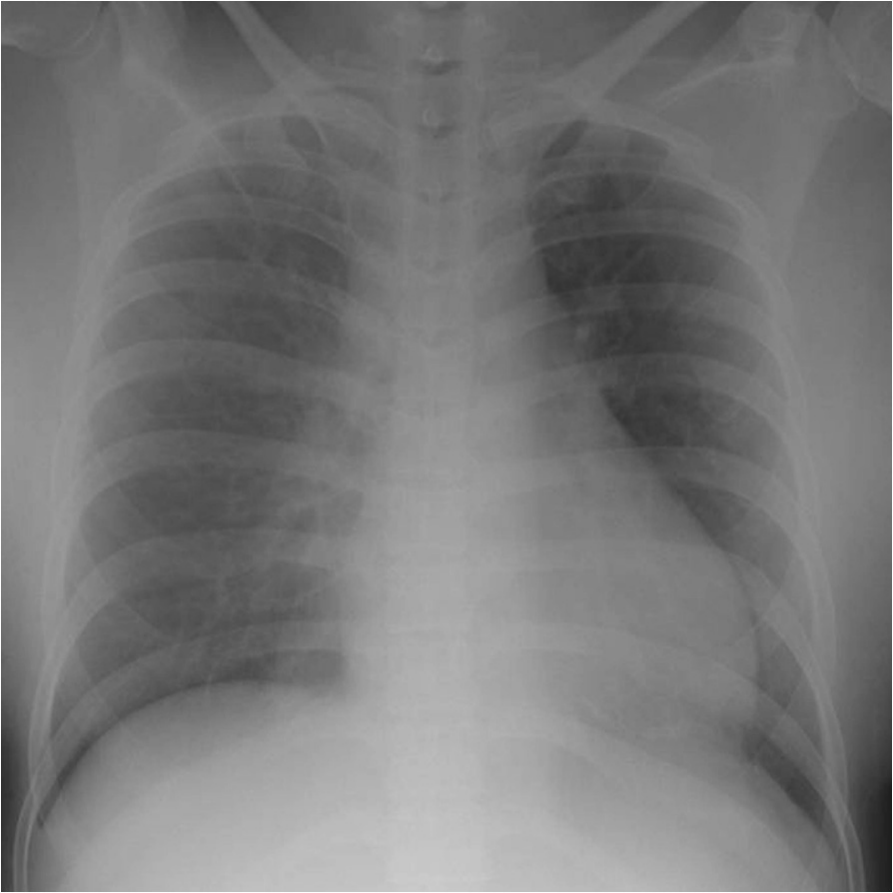
**Chest radiography on admission.** Taken in the semi-sitting position. Shows cardiomegaly (cardiothoracic ratio, 55%), reduced radiolucency of both lung fields, and prominent pulmonary vascular markings.

**Figure 2:**
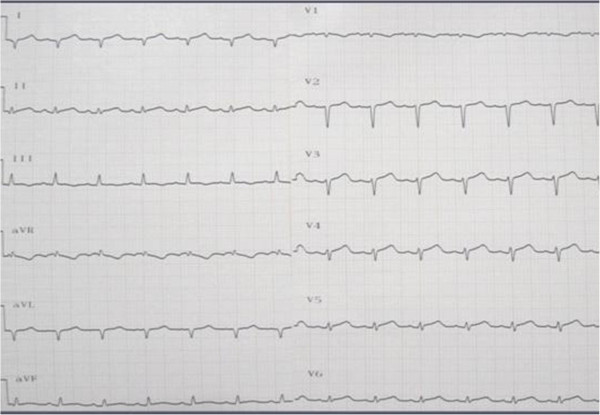
**Electrocardiogram taken at the first visit.** Sinus tachycardia (heart rate, 140/minute), ST elevation in leads I, II, III, aVL, aVf, and V3 to V6, and low QRS voltage in the limb leads.

Chest radiography (Figure 1) revealed cardiomegaly (cardiothoracic ratio, 55%), reduced radiolucency of both lung fields, and prominent pulmonary vascular markings. An electrocardiogram obtained during the first visit (Figure 2) showed sinus tachycardia (heart rate, 130/minute), right axis deviation, QS pattern in leads I and aVL, ST elevation in leads II, III, aVf, and V3 to V6, and a low QRS voltage in the extremity leads.

**Figure 3:**
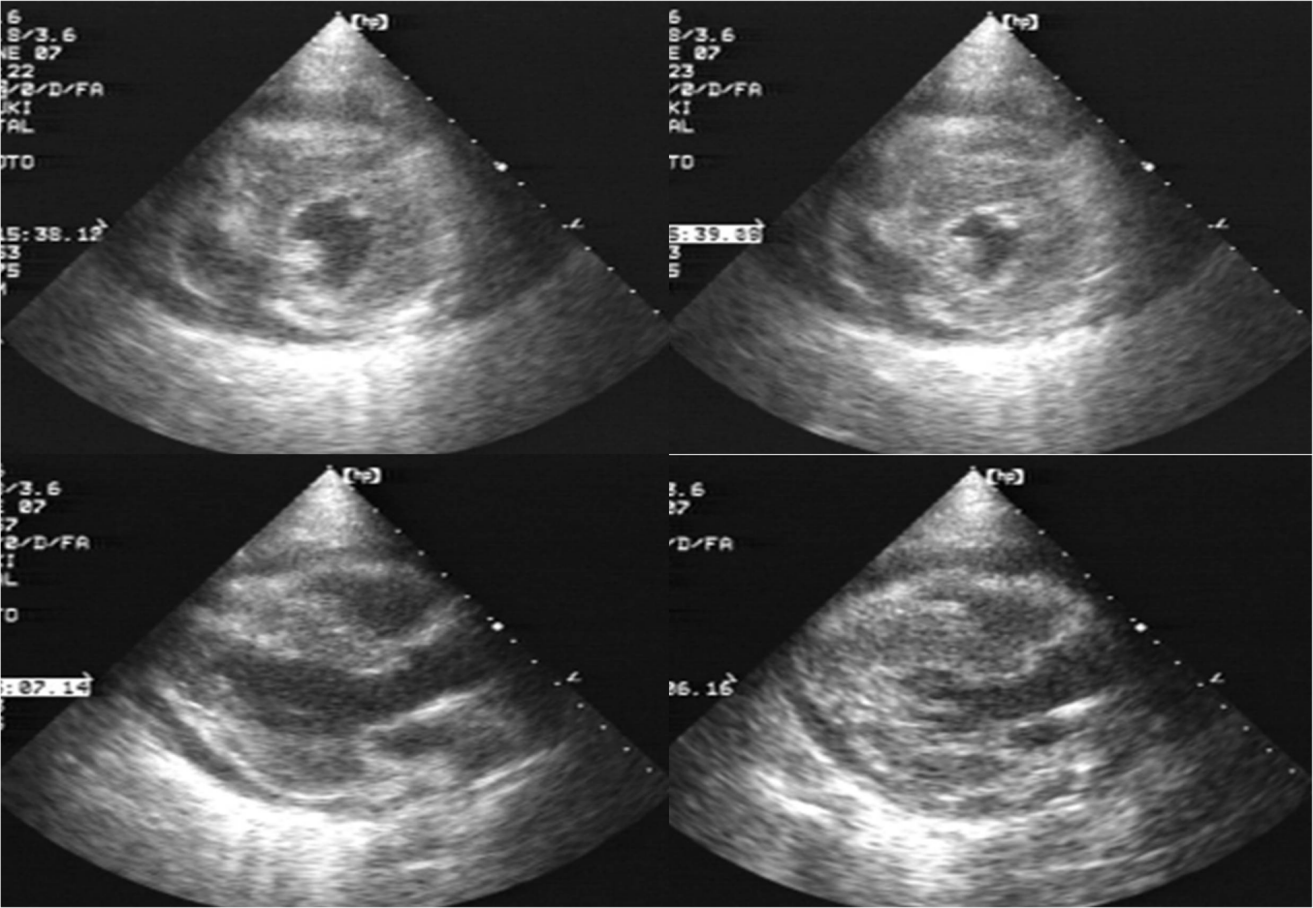
**Echocardiogram on admission.** Long axial view (left) and short axial view (right) obtained via a parathoracic approach. Almost full-circumferential thickening of the left ventricular wall and massive pericardial effusion are noted.

Transthoracic echocardiography (performed via a parathoracic approach) on admission revealed massive pericardial effusion with evidence of collapse of the right atrium and ventricle. The left ventricular wall showed almost full-circumferential thickening (15mm) and diffusely diminished wall motion (ejection fraction 45%, Figure 3). Other abnormalities noted included left ventricular diastolic dysfunction (left ventricular inflow waveform, E:A ratio 0.9), dilatation of the inferior vena cava (diameter, 19mm), tricuspid regurgitation (pressure gradient, 25mmHg), and mild pulmonary hypertension (estimated pulmonary arterial pressure, 35mmHg).

The laboratory test data on admission were as follows: peripheral blood white blood cell count, 21700/mm^3^ (segmented neutrophils 77%, lymphocytes 17%, eosinophils 1%, mononucleated cells 5%); serum C-reactive protein, 2.3mg/dL; serum brain natriuretic peptide (BNP), 610pg/mL; serum creatine kinase (CK)-MM and CK-MB, 202 and 22mg/dL, respectively; test for troponin T, positive. Antiparasitic antibodies and autoantibodies, including antinuclear antibody, were negative.

After admission to the intensive care unit under the clinical diagnosis of acute myopericarditis, she was treated with oxygen supplementation and diuretic therapy. In addition, use of the uterine relaxant isoxsuprine was initiated in consultation with an obstetrician, because she was 14 weeks pregnant. Myocardial biopsy was skipped in order to avoid exposure of the patient and the fetus to radiation and stress. Soon after admission, however, the systolic blood pressure dropped to the order of 70mmHg. Therefore, pericardial drainage was performed for relief of the cardiac tamponade; a yellowish transparent fluid was aspirated at a volume of about 300mL, and the systolic blood pressure rose to about 100mmHg. Thereafter, intravenous antibiotics were initiated. The pericardial effusion had a high specific gravity and was exudative in nature, containing a large number of eosinophils (69%). In addition, hematological examination on hospital day 3 revealed a slight increase in the absolute peripheral blood eosinophil count and elevation of the serum immunoglobulin E level to 1766IU/mL. She was clinically diagnosed with eosinophilic myopericarditis, and the patient was started on oral prednisolone (PSL) therapy at the dose of 30mg/day (0.5mg/kg per day), taking the risks of prolonged decrease in maternal cardiac function and the adverse effects of PSL into account. The patient showed dramatic improvement of the clinical symptoms following the start of the treatment with PSL. The peripheral blood eosinophil count, which had temporarily exceeded 5000/mm^3^, decreased again on hospital day 14, accompanied by a return of the cardiac function to normal (ejection fraction, 60% and left-ventricular wall thickness, 11mm). BNP and CK-MB levels improved to normal range on hospital day 20. The PSL dose was reduced to 20mg, and the patient was discharged from the hospital in a stable clinical condition on hospital day 22 and followed up thereafter as an out-patient. Measurement of viral antibody titers (coxsackievirus, echovirus, and so on) in paired sera revealed no significant elevation of the titers of any of the antibodies.

During the out-patient management, the PSL dose was gradually reduced, without relapse of symptoms. On day 63 after the diagnosis of the disease, oral PSL therapy was discontinued. Although the patient had to be hospitalized for approximately 1 week because she temporarily suffered from pregnancy-induced hypertension, the clinical course was favorable overall, and the patient delivered a healthy baby at the gestational age of 41 weeks.

## Discussion

Eosinophilic myopericarditis is believed to be caused by cytotoxic substances, such as eosinophil cationic protein and major basic protein present in the granules of eosinophils [4]. The diagnosis is established by myocardial biopsy. Eosinophilic myopericarditis may be divided etiologically into three types: (1) parasitic infestation, (2) cardiomyopathy due to chronic eosinophilia as a component of the hypereosinophilic syndrome or Churg–Strauss syndrome and (3) transient and temporary acute myopericarditis due to hypersensitivity which is generally caused by exposure to irritants such as drugs and allergens [1,5,6]. In our patient, although we can rule out parasitic infestation and drug reaction, the role of pregnancy in the pathogenesis of this disease could not be ruled out. According to our literature search, we could not find a relationship between eosinophilia and pregnancy. However, the course of the disease was transient and favorable; thus, the disease was thought to be the acute hypersensitivity type. The clinical course of acute eosinophilic myocarditis, with the exception of fulminant type, is generally transient and its prognosis is good. However, a few cases had major basic protein expression in myocardial biopsy specimens longitudinally after the onset of the disease, therefore, we should cautiously follow up even after alleviation of the acute-phase inflammation [2].

Abundant accumulation of eosinophils in the target organ was noted on admission, although no significant increase in the peripheral blood eosinophil count was noted. One possible explanation for the accumulation of eosinophils in the target organ that has been proposed is that some stimuli induce migration of eosinophils towards the target organ, but the formation of eosinophils in the bone marrow cannot compensate for this change, resulting in a transient paradoxic eosinopenia. Later, eosinophil formation in the bone marrow continues even after tissue infiltration by eosinophils has decreased, resulting in peripheral blood eosinophilia [7]. Although inhalation of the antigen might cause the accumulation of eosinophilic leukocytes via inteleukin-5 in lung disease [8], the precise mechanism of accumulation of eosinophils in the myocardium preceding the increase in the peripheral blood eosinophil count remains unknown. The symptoms of eosinophilic myocarditis are akin to those of viral myocarditis. In the early stages of eosinophilic myocarditis, peripheral blood eosinophilia is sometimes absent [9]. Therefore, it is desirable to check the blood cell counts at intervals of 2 to 3 days when myocarditis is suspected.

Steroids have been shown to be effective in the treatment of eosinophilic myocarditis [1,5]. In regard to the use of steroids during pregnancy, however, animal studies have shown an increased death rate of the fetus following administration of high PSL doses and an increased risk for the development of cleft lip following the use of PSL, even at ordinary doses, during the first trimester of pregnancy [3,10]. Steroid therapy is certainly acceptable in patients with severe disease, as in the case of our patient who presented with cardiac tamponade, because maternal low blood pressure caused by cardiac dysfunction and hypercytokinemia can adversely affect the continuation of pregnancy and the mother’s prognosis. However, unexpected outcomes may be encountered with the use of steroids and cardiac biopsy using X-ray during pregnancy may lead to unexpected outcomes, since adequate clinical data related to its use during pregnancy is still not available [3]. A typical myopericarditis pattern on T2 and late gadolinium-enhanced sequences of cardiac magnetic resonance imaging can be useful in the case of pregnancy [11]. Both patients and their family members tend to be anxious about the use of these drugs, especially during pregnancy. Therefore, we should explain the risks and benefits of this therapy to patients and their family members prior to the start of this treatment.

In a previous report of a patient with eosinophilic myocarditis developing at 24 weeks of pregnancy, thickening of the ventricular septum was observed, with subsequent increase in the peripheral blood eosinophil count, which clinched the diagnosis. The disease was mild in that case, and resolved in response to symptomatic therapy with verapamil, furosemide, and so on [12]. In our present patient, the disease was severe, and the patient presented with cardiac tamponade. Treatment was difficult because only a limited number of drugs could be used because the patient was in the early stages of pregnancy. However, appropriate pericardial drainage and PSL therapy resulted in early alleviation of the condition, which allowed continuation of the pregnancy and successful child delivery. This is a type of case as yet unrepresented the literature, which may therefore prove clinically informative.

## Conclusions

We encountered a patient with myopericarditis in the early stage of pregnancy who improved rapidly with pericardial drainage and PSL therapy, and successfully delivered a normal full-term infant; the diagnosis was made in the early stage of the disease, based on the detection of an abundance of eosinophils in the pericardial effusion preceding the subsequent development of peripheral blood eosinophilia.

## Consent

Written informed consent was obtained from the patient (who also served as legal guardian for the baby) for publication of this case report and any accompanying images. A copy of the written consent is available for review by the Editor-in-Chief of this journal.

## Competing interests

None of the authors have any conflict of interest to report.

## Authors’ contributions

All authors except for TK worked in the ward of Takatsuki General Hospital and conferred on the treatment and patient’s condition. The data were analyzed and interpreted mainly by HT and TK. All authors read and approved the final manuscript.
